# The AUXIN BINDING PROTEIN 1 Is Required for Differential Auxin Responses Mediating Root Growth

**DOI:** 10.1371/journal.pone.0006648

**Published:** 2009-09-24

**Authors:** Alexandre Tromas, Nils Braun, Philippe Muller, Tatyana Khodus, Ivan A. Paponov, Klaus Palme, Karin Ljung, Ji-Young Lee, Philip Benfey, James A. H. Murray, Ben Scheres, Catherine Perrot-Rechenmann

**Affiliations:** 1 Institut des Sciences du Végétal, CNRS UPR2355, Université Paris Sud Orsay, Gif sur Yvette, France; 2 Institut für Biology II – Zellbiologie Universität Freiburg, Freiburg, Germany; 3 Department of Forest Genetics and Plant Physiology, Umeå Plant Science Centre, Sveriges Lantbruksuniversitet, Umeå, Sweden; 4 Department of Biology, Duke University, Durham, North Carolina, United States of America; 5 Institute of Biotechnology, University of Cambridge, Cambridge, United Kingdom; 6 Department of Molecular Cell Biology, Utrecht University, Utrecht, The Netherlands; University of Melbourne, Australia

## Abstract

**Background:**

In plants, the phytohormone auxin is a crucial regulator sustaining growth and development. At the cellular level, auxin is interpreted differentially in a tissue- and dose-dependent manner. Mechanisms of auxin signalling are partially unknown and the contribution of the AUXIN BINDING PROTEIN 1 (ABP1) as an auxin receptor is still a matter of debate.

**Methodology/Principal Findings:**

Here we took advantage of the present knowledge of the root biological system to demonstrate that ABP1 is required for auxin response. The use of conditional ABP1 defective plants reveals that the protein is essential for maintenance of the root meristem and acts at least on the D-type CYCLIN/RETINOBLASTOMA pathway to control entry into the cell cycle. ABP1 affects PLETHORA gradients and confers auxin sensitivity to root cells thus defining the competence of the cells to be maintained within the meristem or to elongate. ABP1 is also implicated in the regulation of gene expression in response to auxin.

**Conclusions/Significance:**

Our data support that ABP1 is a key regulator for root growth and is required for auxin-mediated responses. Differential effects of ABP1 on various auxin responses support a model in which ABP1 is the major regulator for auxin action on the cell cycle and regulates auxin-mediated gene expression and cell elongation in addition to the already well known TIR1-mediated ubiquitination pathway.

## Introduction

The plant hormone auxin plays crucial roles in plant development. While one F-box protein mediated signal transduction route has been discovered, mechanisms of auxin signalling are still partially unknown. Effects of differential accumulation of auxin have been closely analyzed in Arabidopsis roots, where auxin mediates stem cell specification, maintenance of the root meristem, patterning and growth. At the cellular level, auxin is interpreted differentially in a tissue- and dose-dependent manner. Auxin concentrations that promote cell expansion in shoot tissues inhibit cell elongation and promote cell division in roots suggesting that in addition to the importance of auxin distribution and local auxin concentration, differences of cell responsiveness also play critical roles. In the presence of auxin, Aux/IAA transcriptional repressor proteins are recruited by the F-box protein TIR1 within the SCF^TIR1^ complex, polyubiquitinylated and degraded *via* the 26S proteasome [1], [2]. TIR1 binds auxin and acts as an auxin receptor mediating rapid Aux/IAA protein degradation and subsequent Auxin Response Factor (ARF)-dependent activation of transcription [3], [4], [5]. Auxin responses, however, involve another putative auxin receptor, the AUXIN BINDING PROTEIN1 (ABP1) [6]. This protein was isolated based on its capacity to bind auxin and is involved in a set of early auxin responses such as rapid activation of ion fluxes at the plasma membrane [6]. Previous efforts to characterize ABP1's role during plant development have been hampered by the embryo-lethality of the null *abp1* mutant in Arabidopsis [7]. Developmental map of gene expression in Arabidopsis revealed that *ABP1* (At4g02980) exhibit a fairly constant expression in almost all tissues throughout vegetative plant development suggesting that its role is not restricted to embryo development (supporting figure S1) [8], [9]. Using conditional ABP1 Arabidopsis lines, we recently showed that ABP1 is required for post-embryonic shoot development acting on various cellular responses in a context-dependent manner [10]. It remains, however, unknown whether auxin is required for ABP1-driven downstream responses and what is ABP1's role in plant root growth.

Primary root growth is sustained by cell division within the root meristem, which ensures the continuous production of new cells that elongate and differentiate. Accumulating evidence indicates that auxin controls cell identity, cell division and cell expansion in a dose-dependent manner [11], [12], [13], [14]. The primary root exhibits a longitudinal gradient of cell differentiation overlapping an instructive gradient of auxin [11]. Thus, we used the Arabidopsis root as a model to dissect the role of ABP1 in the auxin mediated control of growth.

## Results

### ABP1 is essential for root growth

**Figure 1:**
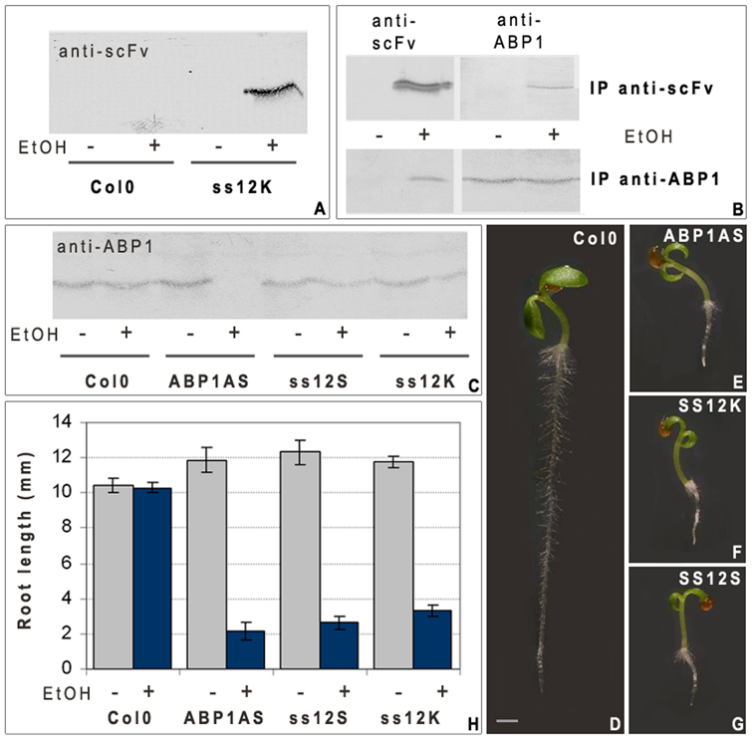
Inactivation of ABP1 affects post-embryonic root growth. A, Immunodetection of scFv using anti-Etag antibody in Col0 and SS12K induced by ethanol or not. The recombinant antibody is detected in samples of ethanol induced SS12K. B, Western blot analysis of scFv and ABP1 on immunoprecipitates using anti-ABP1 mAb34 antibody, which recognizes an ABP1 epitope distinct from scFv12, or using anti-Etag scFv as indicated. ABP1 was co-immunoprecipitated with scFv12/E-Tag and reciprocally scFv12 was co-immunoprecipitated with ABP1/mAb34 in seedling samples of ethanol induced SS12K. C, Western blot analysis of ABP1 protein accumulation in Col0, ABP1-AS, SS12S, and the SS12K, induced or non induced using anti-ABP1 mAb34 antibody. The protein is not detected in root samples of ethanol induced ABP1AS whereas it is detected in controls and scFv12 producing lines. D–G, Severe inhibition of root growth after inactivation of ABP1 function. 3dpg seedlings treated by ethanol vapour since germination are shown. D,Col0; E, ABP1AS; F, SS12K; G, ss12S. Scale bar 1 mm. H, Root length with or without ethanol induction of Col0, ABP1AS, SS12S and SS12K lines at 3dpg. Error bars represent standard deviation (n>40).

**Figure 2:**
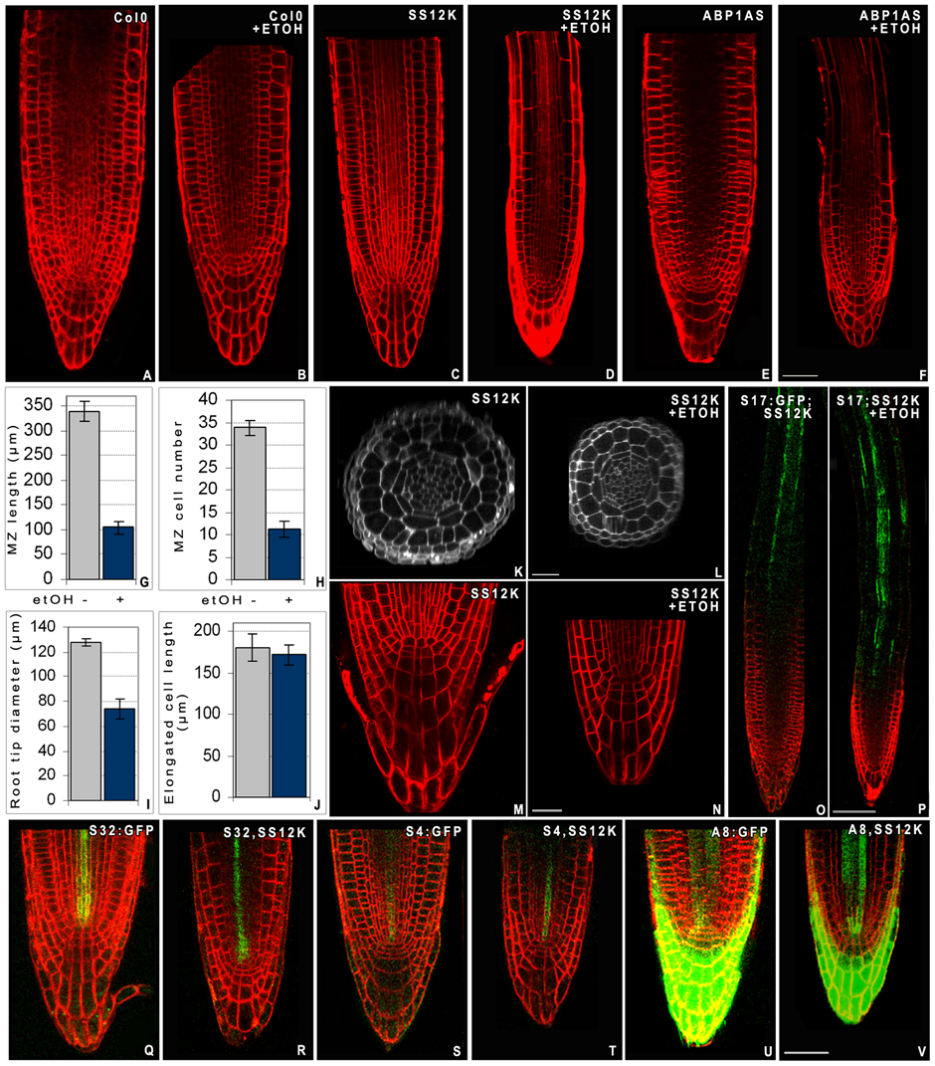
Inactivation of ABP1 leads to consumption of meristematic cells. A–F, Histology of root apex visualized on optical longitudinal sections of living roots stained with FM4-64 (in red). A,B, Col0; C,D, SS12K; E,F, ABP1-AS of 4 day old seedlings grown in the absence (A,C,E) or in the presence of ethanol vapour (B,D,F). Scale bar 40 µm. G–J, Morphometric analysis comparing ethanol induced (blue bars) and non induced (grey bars) ABP1AS at 3dpg. G, meristematic zone length measured from the QC to the end of the lateral root cap; H, cortex cell number in the meristematic zone; I, root tip diameter measured at the end of the lateral root cap; J, epidermal and cortical cell length of differentiated cells, measured after the emergence of root hairs. Standard deviation were calculated from sample number >40. K–L, Optical radial sections of non induced (K) and ethanol induced (L) SS12K roots taken below the end of the lateral root cap. M–N, Close up of the root tip organisation with a focus on the QC and the columella of SS12K not induced (M) or ethanol induced (N). Scale bar 40 µm. O–P, S17:GFP marker AT2G22850 of differentiated phloem pole pericycle (in green) in non induced (O) and induced (P) SS12K seedlings at 4 dpg. Scale bar 80 µm. Q–V, Patterning of cell type specific markers in induced control (Q, S, U) and ABP1-AS (R, T, V) roots. Protophloem S32:GFP AT2G18380 (Q, R); protoxylem S4:GFP AT3G25710 (S, T) and root cap and procambium A8:GFP AT3G48100 (U, V) [16]. Scale bar 40 µm. GFP marker lines [16] were introgressed into SS12K and ABP1AS plants and double homozygous plants were selected from F3 progeny. No significant changes of GFP (in green) expression pattern were detected between ethanol induced or non induced ABP1AS plants or in comparison with reported expression in wild-type background.

To circumvent the embryo-lethality of ABP1 knock-out [7], we used ethanol inducible conditional knock-down plants generated *via* an antisense ABP1 construct to decrease its expression (ABP1AS lines) or *via* cellular immunization to inactivate ABP1 protein through its in vivo interaction with the recombinant antibody scFv12. The latter recognizes a conformational epitope of ABP1 overlapping the auxin binding site (SS12S and SS12K lines) thus impairing the capacity of the protein to bind and respond to auxin [10], [15]. The recombinant antibody was detected in enriched microsomal samples of ethanol induced SS12K (Figure 1A) and we showed by reciprocal co-immunoprecipitation experiments that the scFv12 produced in Arabidopsis interacts with AtABP1 *in vivo* (Figure 1B). ABP1 was still detected in root samples expressing the scFv12 whereas the protein was not detected in induced antisense samples (Figure 1C). At three days post germination (dpg), ethanol induced SS12S, SS12K and ABP1AS plants exhibited similar phenotypes displaying drastic root growth reduction of 60 to 80% compared to ethanol induced control plants (Figure 1D–H). To determine which cellular alterations were responsible for such severe root growth defect, we performed a detailed analysis of SS12K and ABP1AS primary roots. The size of the meristem of ABP1 inactivated roots is about one third of that of controls which correlates with a reduced number of meristematic cells (Figures 2 A–H). Differentiated cortical cells reach a similar length as in control roots (Figure 2J), indicating that longitudinal elongation is not defective in ABP1 inactivated plants. The root diameter is, however, reduced by more than 40% due to decreased radial expansion but the radial tissue organisation inherited from the embryonic root pattern is unaltered (Figure 2I, K, L). Introgression of a series of specific cell type GFP marker lines [16] confirmed maintenance of radial patterns (Figure 2Q–V). At the root apex, a cell layer is missing in both columella and lateral root cap in more then 80% of roots with repressed ABP1 activity (Figure 2M–N). Changes in the longitudinal gradient of root differentiation was confirmed by the use of the S17 GFP marker [16], which is expressed in phloem pole pericycle cells and is detected in the differentiation zone (Figure 2O). After ABP1 inactivation, S17 marker expression is observed at a more distal position, indicating that cells that have left the meristem rapidly begin differentiation (Figure 2P).

### ABP1 controls meristem size

**Figure 3:**
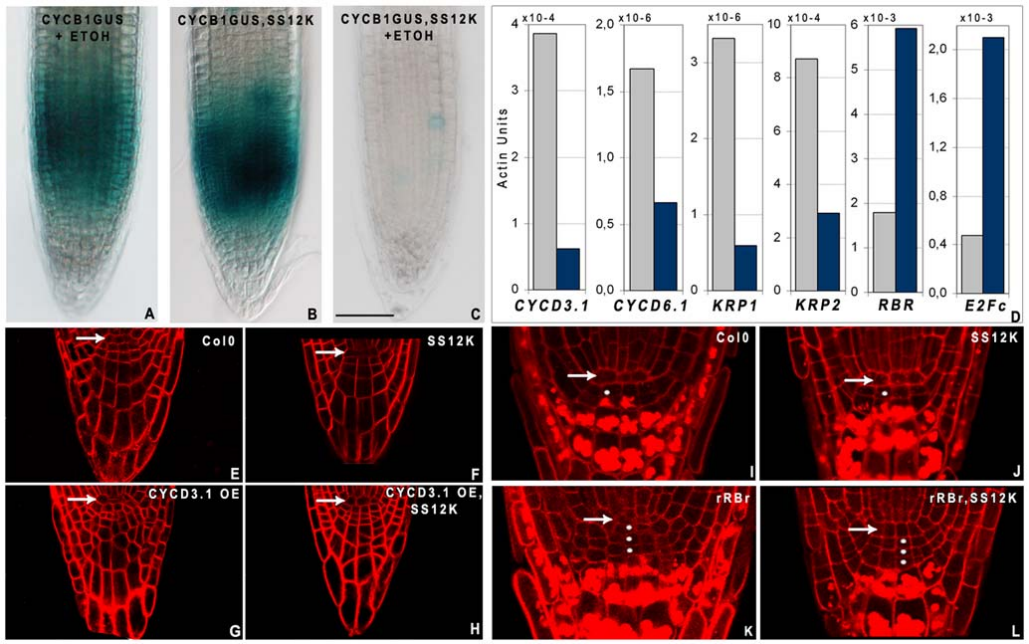
ABP1 inactivation inhibits cell division in root meristem. A–C, Gus staining revealing DboxCYCB1;1-GUS pattern at the root tip of A, ethanol induced wild-type; B, non induced SS12K and C, 24 h ethanol induced SS12K. A strong decrease of DboxCYCB1;1-GUS expression follows inactivation of ABP1. D, Quantitative RT-PCR analysis of core cell cycle markers expression in Col0 (grey bars) and SS12K (blue bars) root seedlings treated for overnight with ethanol vapours. All data were normalized with respect to *ACTIN2-8* and expressed in equivalent *ACTIN* units. E–H, Overexpression of CYCD3.1 restores additional divisions with altered division plane at the columella and lateral root cap in plants inactivated for ABP1. All seedlings were ethanol induced since germination. E, Wt; F, SS12K; G, CYCD3.1OE in Wt background; H, CYCD3.1OE in SS12K background. Living roots stained with FM4-64. I–L, Decreased expression of RBR at the root apex bypasses cell division arrest in the columella stem cells of plants inactivated for ABP1 and leads to the formation of additional stem cell layers. I, Col0 control; J, SS12K; K, *pRCH1:RBR RNAi* line (rRBr) in Wt background; L, rRBr in SS12K background. All seedlings were ethanol induced since germination. Fixed tissues stained with propidium iodide revealing statholiths in differentiated columella cells. Arrows point the QC and dots the columella stem cell layers.

The decrease in root meristem size could result from decreased activity of stem cells, a reduction of division of the daughter cells within the proximal meristem or accelerated consumption of meristematic cells towards elongation and differentiation. To discriminate among these possibilities we first made use of the G2/M phase *pCYCB1::DboxCYCB1;1:UIDA* reporter [17] to evaluate the effect of ABP1 inactivation on the mitotic activity of meristematic cells. After short term ABP1 inactivation, expression of DboxCYCB1;1:GUS is severely reduced (Figure 3A–C), indicating that cells are no longer dividing and also that cells are not arrested in G2 or at the G2/M transition. Expression analysis of G1/S cell cycle markers revealed rapid changes in the mRNA accumulation of various markers after inactivation of ABP1, notably an increase in mRNA accumulation for the RETINOBLASTOMA-RELATED protein (RBR) and the E2Fc transcriptional repressor, and a moderate to strong decrease for Cyclin Dependent Kinase inhibitors (KRPs) and early D-type Cyclins, respectively (Figure 3D). D-type cyclins have been proposed to integrate various signals and to be limiting for the G1 to S phase transition through their interaction with CDK and further phosphorylation of RBR [18]. We hypothesized that the rapid decrease in D-type Cyclin expression and/or the increase in RBR following inactivation of ABP1 could disturb the CYCLIN D/RBR regulatory pathway and thus contribute to the arrest of cell division. We then explored whether compensating for these expression changes by either overexpressing CYCD3.1 or reducing RBR expression would be sufficient to restore cell division (Figure 3E–L). In a wild-type background, increased CYCD3.1 [18] or decreased RBR (rRBr line) [19] do not change root growth rate and meristem size but supernumerary stem cells appear post-embryonically in the columella and lateral root cap (Figure 3E, G, I, K). We crossed these lines with SS12K plants and focused on root stem cells. In ethanol induced SS12K,CYCD3.1OE roots, supernumeray cells sometimes with aberrant cell plate formation are observed as in the CYCD3.1OE (Figure 3G,H), indicating that increased expression fo CYCD3.1 in ABP1 inactivated cells is sufficient to restore cell division in stem cells. Similarly, in ethanol induced SS12K,rRBr roots, additional stem cell layers are generated as in the rRBr control line (Figure 3K, L). Therefore, both overexpression of CYCD3.1 and reduction of RBR bypass the cell cycle arrest mediated by inactivation of ABP1 in root cap stem cells, suggesting that the CYCLIN D/RBR pathway is operating downstream of ABP1. Future investigations will be necessary to identify the signaling pathway connecting ABP1 to the G1/S regulatory complex and to provide a comprehensive view of the role of ABP1 and auxin in cell division.

**Figure 4:**
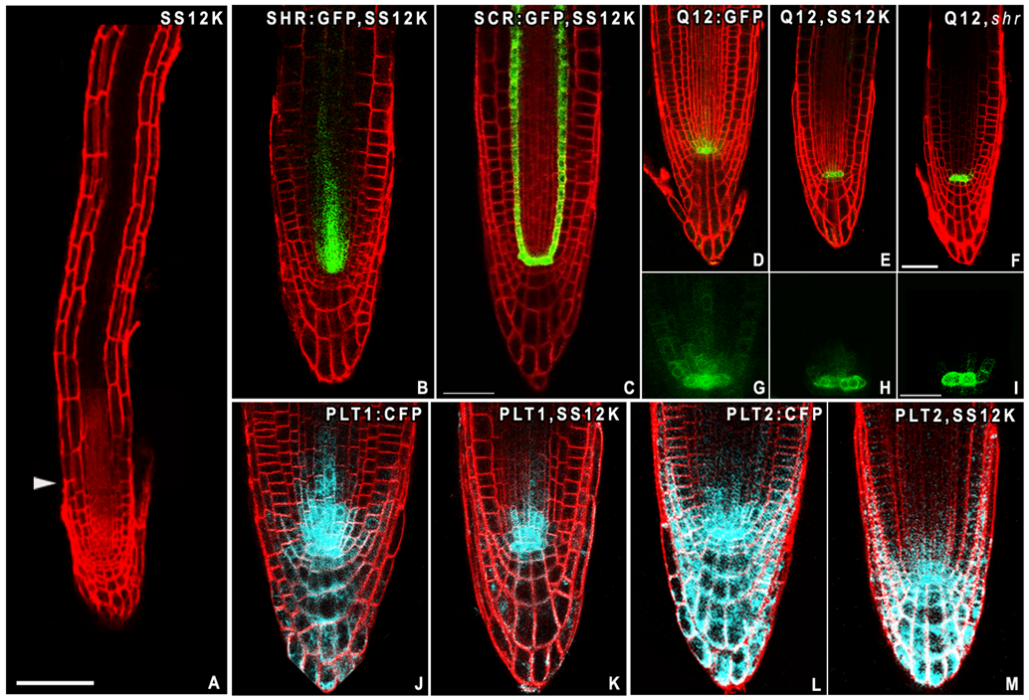
Post-embryonic ABP1 inactivation causes subtle defects in stem cell maintenance. A, Root phenotype of long-term ABP1 inactivated SS12K plant. Ethanol induction was maintained from germination to 14 dpg. Long cells are observed close to the QC, the elongation zone is absent and the meristem is restricted to five cells. The arrow points the end of the meristematic zone. Scale bar 80 µm. B–C, Unmodified patterning of pSHR:GFP (B) and pSCR:GFP (C) expression in induced ABP1AS at 4dpg. GFP in green, FM4-64 staining in red. D–I, Expression pattern of pQ12:GFP (AT5G17800) in 4 dpg seedlings ethanol induced Col0 (D,G), ABP1AS (E,H) and *shr2* (F,I) showing strong reduction in the stele stem cells of both ABP1AS and *shr2*. J–M, Expression pattern of *PLETHORA* using full-size promoters driven CFP reporter (in blue) in 4 dpg seedlings ethanol induced since germination. *pPLT1:CFP* in Col0 (J) and SS12K (K); *pPLT2:CFP* in Col0 (L) and in SS12K (M). Inactivation of ABP1 provokes a reduction of the overall expression area of *PLT*.

The roots of SS12K seedlings inactivated for ABP1 for up to 15 days exhibit elongated and differentiated cells next to a residual meristem made of 5 to 6 contiguous cells above the QC (Figure 4A). These meristematic cells have been prevented from differentiation even if they have lost the capacity to divide suggesting that they were not competent for elongation and differentiation.

To investigate whether arrest of cell division and reduction of meristem size result from a defect in stem cell activity, we studied expression of genes involved in stem cell specification. Two members of the GRAS family of transcription factors, SHORT-ROOT (SHR) and SCARECROW (SCR) are required for QC identity and are also involved in root radial patterning during embryogenesis [20], [21]. Expression patterns of *SHR* and *SCR* are not altered in SS12K ethanol induced plants (Figure 4B,C). Expression of Q12, a putative transcription factor specifically expressed in the QC and the stele stem cells [16], was maintained in the QC but was consistently decreased or absent from stem cells (Figure 4D, E, G, H). Interestingly, similar results were performed when this marker was introduced into the *shr-2* mutant (Figure 4F,I). The expression of *SHR* and *SCR* is however not modified in roots inactivated for ABP1 and no differentiation of the columella stem cells is observed, contrary to what was seen in the *shr* mutant, suggesting that ABP1 does not act on SHR.

**Figure 5:**
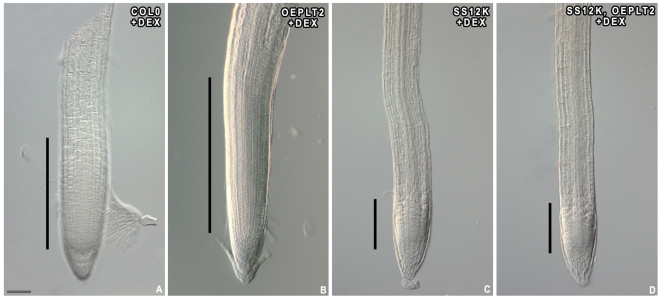
Overexpression of PLT2 does not reactivate cell division in roots inactivated for ABP1. Ethanol induced 35S-PLT2-GR roots in Col0 (A–B) and in SS12K (C–D) without dexamethasone (A,C) and after 24 h application of 10 µM dex (B,D). Overexpression of PLT2 cannot bypass cell cycle arrest mediated by ABP1 inactivation. Vertical bars represent meristem length. Scale bar 40 µm.

Genes of the *PLETHORA* family, encoding AP2-domain transcription factors, are also essential for root stem cell maintenance and are related to auxin action. PLTs were recently revealed to control distinct aspects of root development in a dose-dependent manner [22], [23]. The general patterning of *PLTs* was unchanged in roots inactivated for ABP1 but the overall area of expression was consistently reduced compared to controls (Figure 4J–M) suggesting that the resulting PLT activity is limited to fewer cells, around the QC. To explore whether increased levels of PLT would be sufficient to either restore cell division within the meristem or to delay differentiation of meristematic cells, we overexpressed PLT2-GR in SS12K plants [22]. Dexamethasone-induced PLT2 expression in ABP1 inactivated roots did not reactivate cell division (Figure 5), coherent with previous data supporting that PLT overexpression sustains cell division only in cells that still have the capacity to divide [22]. PLT2 overexpression at an early stage of ABP1 inactivation (before most meristematic cells have elongated) however inhibits cell expansion of cells from the basal meristem and the transition zone, thus maintaining meristem size (not shown). This observation confirms the dose-dependent PLT requirement for the transition between the meristematic and the elongation zones and suggests that PLT2 is acting downstream of ABP1 to facilitate elongation. All these results suggest that ABP1 activity defines a zone of competence for PLT activity essential to control the transition between the meristem and the elongation zone.

### ABP1 mediates auxin responsiveness

Observation of root growth defects resulting from ABP1 inactivation reinforces the correlation between the protein and auxin. These defects could, however, result from various initial defects including an alteration of auxin content, an alteration of auxin transport or a shift in auxin sensitivity.

**Figure 6:**
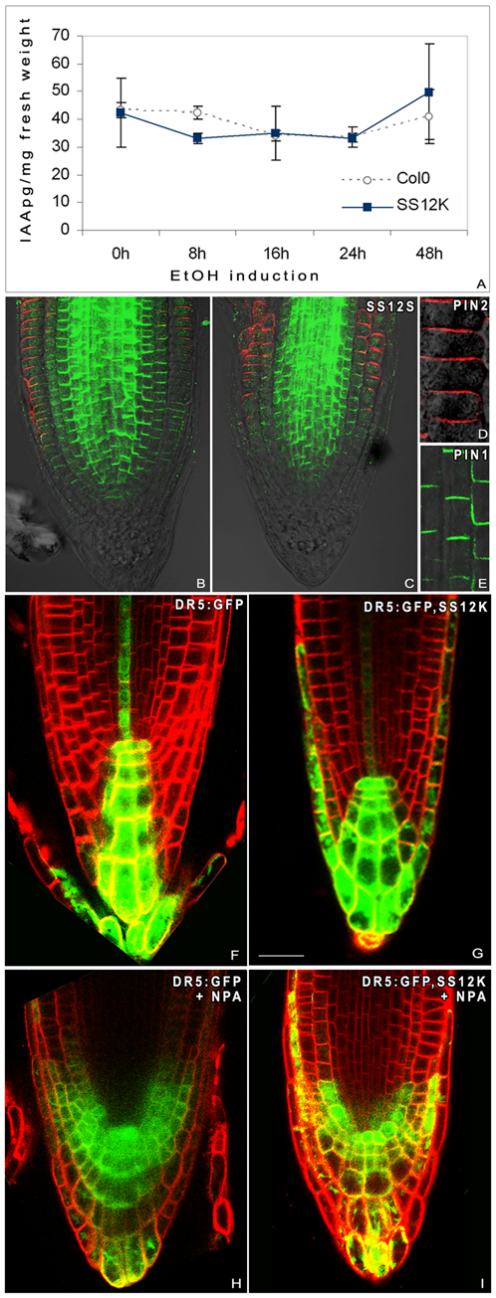
ABP1 does not affect directly auxin content and auxin transport in roots. A, Analysis of IAA accumulation in Col0 and SS12K roots after various times of ethanol induction as indicated. Free IAA content was measured in root samples of 4 dpg control and SS12K lines. Inactivation of ABP1 does not affect the auxin content of root tissues. B–E, Immunolocalization of PIN1 (in green) and PIN2 (in red) in 4dpg Col0 (B) and SS12S (C–E) seedlings induced for 48 h with ethanol. D, E Inserts are enlargements showing apical and basal localisation for PIN1 and PIN2, respectively. No significant difference of PIN localisation is observed. PIN1 is located at the apical and lateral sides of stele cells whereas PIN2 is located at the basal side of epidermal cells and apically and laterally in cortical cells. F–I, DR5:GFP reporter expression pattern in 4dpg Col0 (F,H) and SS12K (G,I) ethanol induced seedlings grown in the absence (F–G) and in the presence of 10 µM NPA (H–I). Scale bars 40 µm. DR5:GFP visualizes the auxin response maximum and shows similar expression in roots inactivated for ABP1 and controls with a maximum in the QC, columella stem cells and differentiated cells and within the proximal meristem in some provascular cells. In the presence of 10 µM NPA, the DR5:GFP maximum was expanded laterally and shifted back in epidermis and cortical cells from the proximal meristem in both samples (H–I).

To discriminate among these hypotheses, we first measured free IAA content in root samples after various times of ethanol induction. No significant differences were observed over a period of 48 h of ABP1 inactivation (Figure 6A), suggesting that no global change in auxin content has occured within this time frame. Second, to assess whether the observed root phenotype is due to more subtle changes in auxin distribution, we performed immunolocalization of PIN1 and PIN2 proteins. PIN proteins have been reported to control root meristem size through their tight control of auxin redistribution at the root apex [11], [24]. Two days after inactivation of ABP1, PIN protein localization was not significantly modified (Figure 6B–E). Upon prolonged inactivation, the signal decreases in correlation with cell differentiation especially for PIN2 (not shown). Thus, changes in PIN expression are likely to be a secondary consequence of ABP1 inactivation's effect on cell differentiation rather than a primary effect. As another indicator of auxin redistribution in induced SS12K roots, we monitored expression of the auxin responsive reporter DR5:GFP [25] in the absence and in the presence of the auxin transport inhibitor 1-N-naphthylphthalamic acid (NPA). We found that inactivation of ABP1 has no significant effect on the expression of the DR5:GFP reporter at the root apex (Figure 6F–G) and that NPA was able to disturb the auxin maxima independently of ABP1 activity (Figure 6H–I). This data indicates that auxin transport is still efficient in roots inactivated for ABP1 revealing that the carriers are functional. Taken together these results show that ABP1 is not essential to maintain PIN activities or the auxin gradient in the root apex.

We thus investigate the third hypothesis that ABP1 contributes to auxin responsiveness. We analysed the effect of exogenous auxin on the major auxin responses: cell division, cell elongation and gene expression. We first attempted to reactivate cell division in the root meristem by treatment with various concentrations of exogenous auxin. Based on DboxCYCB1;1:GUS detection, we found that cell division cannot be reactivated in root meristem of ABP1 inactivated plants whatever the auxin concentration applied (not shown), thus revealing an insensitivity to auxin of the cells towards the response of division. This data confirms that ABP1 exerts a strict control on cell division and it suggests that ABP1 is required for the capacity of meristematic cells to divide in response to auxin.

**Figure 7:**
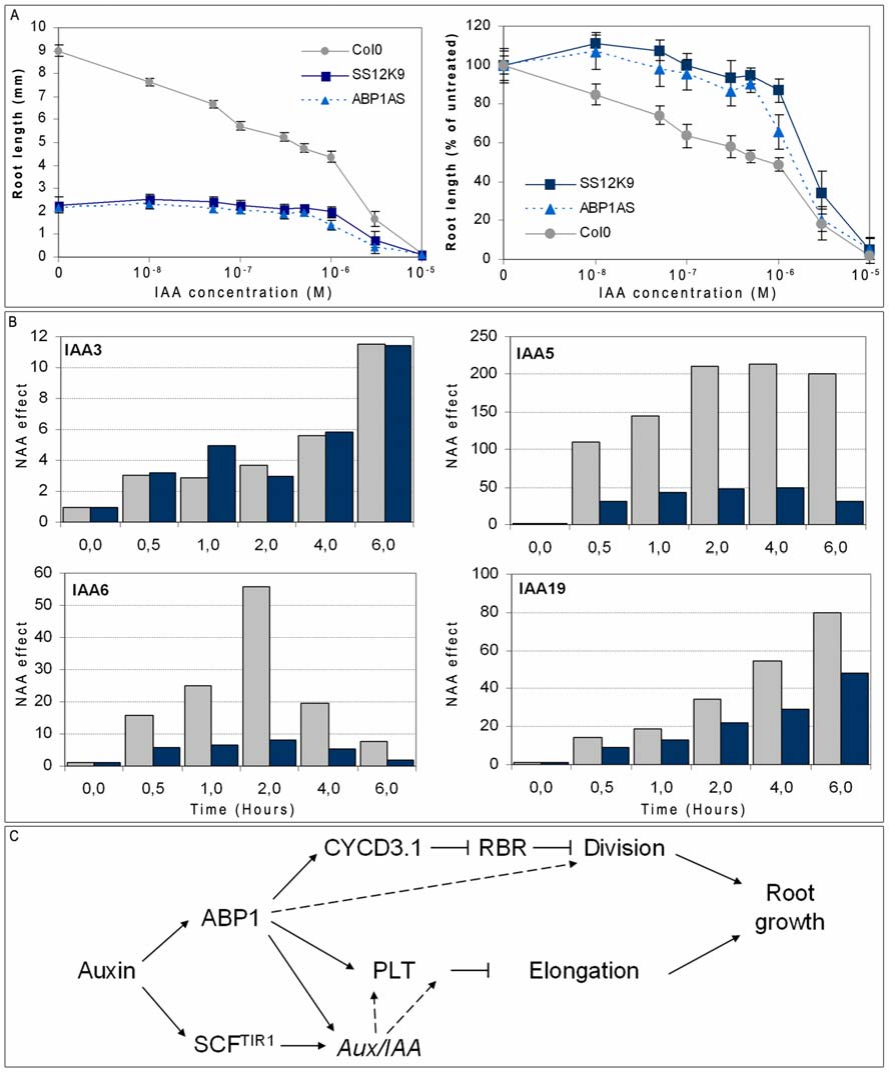
Inactivation of ABP1 impairs auxin responsiveness. A, Auxin dependent root growth curve of ethanol induced Col0, ABP1AS and SS12K. Seedlings were grown in the presence of various concentrations of IAA as indicated and induced with ethanol since germination. On the first panel, root length measurements at indicated IAA concentrations are plotted. On the second panel, data are expressed relative to the root length of each genotype grown without auxin. Standard deviations were calculated from samples >40. B, Kinetic effects of 1 µM NAA treatment on transcript accumulation of *Aux/IAA* genes in roots of overnight ethanol induced control (open bars) and SS12K (grey bars). Data were normalized with *ACTIN2-8* then to the expression level at time zero of auxin application. C, A model for ABP1 mediated auxin responses in roots. The permissive effect of auxin on cell division is dependent on ABP1. In root stem cells, the D-type CYCLIN/RETINOBLASTOMA (RBR) pathway acts downstream of ABP1 and controls the G1/S transition. In meristematic cells, ABP1 might also affect the D-type CYCLIN/RBR pathway but other critical regulators of the G1/S transition phase are dependent on ABP1 activity. ABP1 contributes to the auxin control of cell elongation by modulating a zone of competence for PLETHORA and by acting on the auxin-mediated regulation of Aux/IAA transcriptional repressors. It is worthwhile noticing that expression of *PLTs* was reported to be regulated downstream of ARF transcription factors (Auxin Response Factors) [23] and consequently of Aux/IAAs. ABP1 might act indirectly on PLT *via* Aux/IAAs regulation. It is well established that regulation of gene expression by auxin involves the TIR1 receptor which, within the SCF^TIR1^ E3 ligase, controls the degradation of Aux/IAA repressors[1]. ABP1 and TIR1 might collectively control gene regulation and elongation.

Second, we examined the inhibitory effect of exogenous auxin on root elongation. As shown in figure 7A, there was no effect of auxin on ABP1 inactivated root below 10^−6^M IAA indicating that cell elongation was not reduced (also verified by microscopy). In the same conditions, Col0 plants exhibited 50% inhibition of growth corresponding to the inhibition of cell elongation. For IAA concentrations higher than 10^−6^M, a significant inhibition of root growth was observed despite the initial reduced length of ABP1 inactivated roots. This effect resulted from a reduced elongation of the cells as observed for the control at lower concentrations. Roots inactivated for ABP1 were less sensitive than control to primary root elongation inhibition caused by auxin, indicating that ABP1 is also required for this auxin response. Interestingly, it is not a complete insensitivity to auxin as observed for division but a shift in sensitivity suggesting that ABP1 is likely to contribute to this auxin response together with other regulators.

Third, we analyzed the kinetics of *Aux/IAA* mRNA accumulation in response to auxin in short term ethanol induced control and SS12K lines. Auxin efficiently induced the expression of most *Aux/IAA* genes in root samples with minor changes in comparison to controls as illustrated for *IAA3* (out of 14 genes tested) with the notable exception of *IAA5*, *IAA6* and *IAA19* (Figure 7B), three genes belonging to the same clade of *Aux/IAA*s [26]. Auxin responsiveness for these *Aux/IAAs* was reduced by 4 to 5 fold indicating that, in roots, ABP1 is somehow required for optimal gene response to an auxin stimulus. *Aux/IAAs* genes are submitted to various combinatorial regulations which are far to be elucidated for each member of the *Aux/IAA* gene family. The differential effect of ABP1 on the auxin responsiveness of various *Aux/IAAs* in roots illustrates the complexity of the regulatory pathway controling their expression and indicates that ABP1 is involved.

## Discussion

Our data show that ABP1 is implicated to various degrees in the control of auxin responses mediating root growth, especially cell division, cell elongation, and gene expression. ABP1 is essential to maintain the mitotic activity of meristematic cells and stem cells (Figures 2,3). This critical control of cell division in roots confirms previous data obtained on BY2 cell suspension [27], embryo [7] and shoot tissues [10] and reveals the general nature of the control exerted by ABP1 on cell division. Interestingly, this effect is consistent with auxin's permissive role in cell division. Based on analysis of root stem cells, ABP1 affects the D-type CYCLIN/RBR regulatory pathway (Figure 3). Although the molecular link between ABP1 and these cell cycle regulators may not be direct, it is clear that ABP1 is essential for the regulation of the G1/S transition. Within the CYCLIN D/RBR pathway there are multiple potential targets, amongst which CDK inhibitors (KRPs) and E2F transcriptional factors are additional relevant targets. For example, E2Fc has been reported to negatively affect cell division [28] and we cannot exclude that the increased accumulation of E2Fc mRNAs in ABP1 inactivated seedlings also contributes to the inhibition of cell division. Overproduction of E2Fc was shown to inhibit cell proliferation [28], [29]. The E2Fc protein is however submitted to rapid degradation in an ubiquitin dependent manner and changes at the RNA level might not reflect the amount of protein. Protein turnover of E2Fc, as well as activator E2Fb which has been shown to be stabilised in response to auxin in BY2 cells [30], will merit investigation in plants inactivated for ABP1.

The role of ABP1 is however not restricted to the auxin control of cell division, as our results clearly show that the protein is implicated in the auxin regulation of cell elongation and gene expression (Figure 7). By conferring on the root cells a relative sensitivity to auxin, ABP1 contributes to the regulation of cell behavior at the transition zone, likely contributing to deciphering the gradient of auxin [11], [24], [31] and the cross-talk between auxin and cytokinin [32], [33]. It is tempting to hypothesize that the effect of ABP1 on PLT gradient leads to the switch between division and elongation that occurs in the transition zone. Interestingly, cell elongation in roots is inhibited by auxin and occurs as auxin content decreases, whereas cell expansion in shoot tissues is promoted by auxin. Importantly, the differential effect of ABP1 on cell elongation in roots and expansion in shoots [10], [34] perfectly matches known differential effects of auxin on both tissues.

A differential auxin response is also observed on gene expression. ABP1 is required for auxin induced expression of a subset of *Aux/IAA* genes in roots (Figure 7B) whereas we previously observed enhanced auxin responses for the same *Aux/IAA* genes and others in shoot tissues of ABP1 inactivated plants [10], indicating that the fine tuning exerted by ABP1 on the regulation of gene expression differs between root and shoot. Expression of *Aux/IAAs* in response to auxin is known to be mediated by the E3 ubiquitin ligase complex SCF^TIR1^, where the F-box and auxin receptor TIR1 recruits the Aux/IAA repressor protein for ubiquitination and further degradation by the 26S proteasome [1]. Little is known relative to a differential regulation of this mechanism to sustain differences of tissue sensitivity to auxin [35] and our data suggest that ABP1 is involved in this process. ABP1 sits at the plasma membrane, where its role of auxin receptor transducing at least part of the auxin signal was initially demonstrated [2], [6] whereas TIR1 and Aux/IAA substrates are mainly located in the nucleus, thus physical interaction between these proteins is highly unlikely. Multiple signaling components such as MAP kinases [36], [37], IBR5 protein phosphatase [38], [39], phospholipase A2 [40], [41] and RAC GTPases [42], [43] have been reported to be involved in auxin signaling yet have not been implicated in the short auxin signaling SCF^TIR1^ pathway. They are possible candidates mediating ABP1 action on downstream targets. How these regulators interact with each other to mediate ABP1 and auxin dependent gene expression remains to be determined. As only a subset of *Aux/IAA* genes is affected by ABP1 inactivation, we can hypothesized that either ABP1 acts on other transcription factors co-regulating expression of these genes (as Myb77 [44]) independently of TIR1 or ABP1 somehow alters the relative affinity of Aux/IAA and ARF interaction which governs their expression thus interfering with the SCF^TIR1^ pathway. Elucidating the molecular basis of the cross-talk between ABP1-mediated responses and the SCF^TIR1^ pathway will be one of the important challenges for the coming years, in particular the relative positioning of ABP1 and TIR1 in the regulation of gene expression will shed new lighting on the complex network of auxin signalling.

In conclusion, it appears that ABP1 and TIR1 collectively contribute to mediate auxin responses. Based on the data presented here and previous reports (reviewed by [1], [2]) we propose as a working model that ABP1 shares auxin regulation of gene expression and control of cell elongation with the TIR1 pathway whereas ABP1 is the master regulator for the auxin control of cell division (Figure 7C).

## Materials and Methods

An ethics statement is not required for this work.

### Plant lines and growth conditions

The Col-0 ecotype of *Arabiodopsis thaliana* was used for construction of all transgenic plants. Markers, mutant, overexpressor and RNAi lines were introduced by crosses in ABP1 conditional lines [10] and double homozygotes selected from F3 progeny were used for all observations. Controls were either wild-type Col0 or plants expressing GUS under the ethanol inducible system (AlcA:Gus) to guarantee that the observed feature is not related to the ethanol system. Lines used were: CYCB1::DboxCYCB1;1:GUS [17], DR5::GUS [45], promoter::GFP lines A8, Q12, S04, S17, S32 [16], SHR::GFP [46], SCR::GFP [47], promoter::CFP lines PLT1, PLT2 [23], *shr-2* [48], 35S::PLT2-GR [22], RCH1::RBR RNAi (“rRBr”) [19] and 35S::CYCD3;1 which is in Landsberg ecotype [49]. Seeds were germinated under sterile conditions on plates containing 1/2 Murashige and Skoog (MS) basal salt mixture, buffered at pH 5.7 with 2.5 mM 2-(N-morpholino) ethanesulfonic acid, and containing 0.9% vitro agar (Kalys, St Ismier, France). For selection of transgenic plants or segregating plants issuing from crosses with marker lines either kanamycin or hygromycin was used. In all cases, plates were incubated in a vertical position at 22°C under constant light at 99 µmol. m^−2^. s^−1^ intensity.

### Ethanol induction and treatments

The ethanol induction was performed as described [10]. Inductions were performed either immediately after stratification of the seeds or after 1 to 4 days of culture for shorter exposure to ethanol vapors as indicated.

For chemical treatments, seedlings were either grown on half MS and transferred on media containing 1 to 100 nM NAA for the indicated time or grown on 10 µM NPA. The root auxin sensitivity assay was performed by growing seedlings on plates containing appropriate concentrations of IAA. The root length was measured after 4 days of growth. Elongation is expressed relative to the mean root elongation of the same genotype on medium without auxin with standard deviation calculated on at least 40 samples each.

### Root imagery

Whole mount microscopic analysis of roots were performed on fresh material stained with 5 µM FM4–64 for 10 min and roots were observed using an inverted confocal microscope TCS SP2 (Leica microsystems, Heidelberg, Germany). Roots of rRBr and rRBr,SS12K plants were fixed on acetic acid and methanol. Periodic acid-Schiff's reagent was used to reveal starch granules and tissues were stained with Propidium Iodide before observation. The pictures were shaped and assembled using Photoshop (Adobe) without treatment. Quantitative measurements were realised with ImageJ.

The β-glucuronisade (GUS) assays were performed as described [50], samples were stained overnight at 37°C. GUS-stained seedlings were observed without clearing with a MULTIZOOM AZ100 microscope (Nikon Corporation Instruments Company, Japan).

### Immunodetection

Immunolocalization was performed on 4 day old seedlings. Immunolocalization in roots was performed as described [51]. Labeling was performed with rabbit anti-PIN1, guinea pig anti-PIN2 and rabbit anti-PIN4 antibodies at 1∶500, 1∶400 and 1∶500 dilutions, respectively. Alexa Fluor 488-conjugated goat anti-rabbit and Alexa Fluor 555-anti-guinea pig secondary antibodies were used at 1∶400 dilution. During the immunolocalization procedures, solutions were changed using a pipetting robot (InsituPro, Intavis Bioanalytical Instruments AG).

### IAA quantification

Root tips were collected from 4 dpg seedlings exposed to ethanol for the indicated time and frozen immediately in liquid nitrogen. The frozen samples were homogenized in 0.5 ml 50 mM sodium-phosphate buffer pH 7.0 containing 0.02% diethyldithiocarbamic acid (Sigma) and 500 pg [^13^C_6_]-IAA (Cambridge Isotope Laboratories, Andover, MA, USA) internal standard for 2 min at a frequency of 30 Hz, using a Retsch MM 301 vibration mill (Retsch GmbH, Haan, Germany) and a 3 mm tungsten carbide bead. The samples were then incubated for 15 min at +4°C under continuous shaking. The pH was adjusted to 2.7 with 1 M HCl, and the samples were purified by solid phase extraction on a 500 mg Isolute C8 (EC) column (International Sorbent Technology), conditioned with 2 ml methanol and 2 ml 1% acetic acid. After sample application, the column was first washed with 2 ml 10% methanol in 1% acetic acid and then eluted with 2 ml 70% methanol in 1% acetic acid. The dried samples were dissolved in 0.2 ml 2-propanol and 1 ml dichloromethane and 5 µl 2 M trimethylsilyl-diazomethane in hexane (Aldrich) was added to methylate the samples. After methylation, the samples were trimethylsilylated and IAA was quantified by gas chromatography-selected reaction monitoring-mass spectrometry as described in [52]. All samples were analysed in triplicates from two biological repeats.

### Real-time RT-PCR analysis

RNA was extracted from roots of 4dpg seedlings treated with 5 µM NAA for 30 min to 6 hours using an Qiagen RNeasy kit and digested with RNAse free DNAse on the column following the manufacturer's instructions (Qiagen S.A., Courtaboeuf, France). First-strand cDNAs were synthesized from 5 µg of total RNA using Superscript II reverse transcriptase according to the manufacturer's instructions. Quantitative RT-PCR analyses were performed using SYBR Green QPCR master mix (Roche) with specific primers as reported [10]. Two biological repeats were analysed in duplicates.

## Supporting Information

Figure S1 Expression pattern of ABP1 in Arabidopsis Expression data were extracted from AtGenExpress developmental series [8]. Absolute values are linearized gcRMA values. Expression of ABP1 (At4g02980) is compared with PIN2 (At5g57090) taken as a root specific gene and with At2g28390, a member of the SAND family, one of the most stable reference gene throughout development [53]. Various root samples are identified using the number of the AtGenExpress sample ID (http://www.weigelworld.org/resources/microarray/AtGenExpress/AtGE_dev_samples.pdf/view).
(1.06 MB PDF)
