# Supplementary material for: The AUXIN BINDING PROTEIN 1 Is Required for Differential Auxin Responses Mediating Root Growth
Source: PLoS One. 2009 Sep 24;4(9):e6648. doi: 10.1371/journal.pone.0006648 (PMC2744284; doi:10.1371/journal.pone.0006648)
Supplement: Figure S1 — Expression pattern of ABP1 in Arabidopsis Expression data were extracted from AtGenExpress developmental series [8]. Absolute values are linearized gcRMA values. Expression of ABP1 (At4g02980) is compared with PIN2 (At5g57090) taken as a root specific gene and with At2g28390, a member of the SAND family, one of the most stable reference gene throughout development [53]. Various root samples are identified using the number of the AtGenExpress sample ID (http://www.weigelworld.org/resources/microarray/AtGenExpress/AtGE_dev_samples.pdf/view). (1.06 MB PDF) [file pone.0006648.s001.pdf]

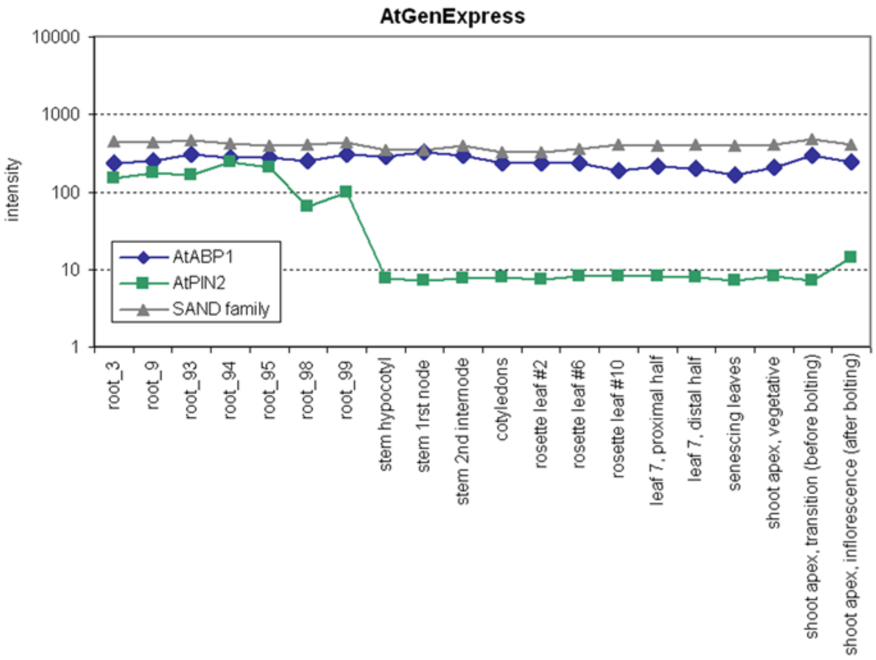

Figure S1 – Expression pattern of ABP1 in Arabidopsis

Expression data were extracted from AtGenExpress developmental series [8]. Absolute values are linearized gcRMA values. Expression of ABP1 (At4g02980) is compared with PIN2 (At5g57090) taken as a root specific gene and with At2g28390, a member of the SAND family, one of the most stable reference gene throughout development [53]. Various root samples are identified using the number of the AtGenExpress sample ID:

(http://www.weigelworld.org/resources/microarray/AtGenExpress/AtGE_dev_samples.pdf/view).
